# Aorto-ventricular tunnel

**DOI:** 10.1186/1750-1172-2-41

**Published:** 2007-10-08

**Authors:** Roxane McKay

**Affiliations:** 1 Division of Cardiovascular Surgery, Le Bonheur Children's Hospital, Memphis, TN 38103, USA

## Abstract

Aorto-ventricular tunnel is a congenital, extracardiac channel which connects the ascending aorta above the sinutubular junction to the cavity of the left, or (less commonly) right ventricle. The exact incidence is unknown, estimates ranging from 0.5% of fetal cardiac malformations to less than 0.1% of congenitally malformed hearts in clinico-pathological series. Approximately 130 cases have been reported in the literature, about twice as many cases in males as in females. Associated defects, usually involving the proximal coronary arteries, or the aortic or pulmonary valves, are present in nearly half the cases. Occasional patients present with an asymptomatic heart murmur and cardiac enlargement, but most suffer heart failure in the first year of life. The etiology of aorto-ventricular tunnel is uncertain. It appears to result from a combination of maldevelopment of the cushions which give rise to the pulmonary and aortic roots, and abnormal separation of these structures. Echocardiography is the diagnostic investigation of choice. Antenatal diagnosis by fetal echocardiography is reliable after 18 weeks gestation. Aorto-ventricular tunnel must be distinguished from other lesions which cause rapid run-off of blood from the aorta and produce cardiac failure. Optimal management of symptomatic aorto-ventricular tunnel consists of diagnosis by echocardiography, complimented with cardiac catheterization as needed to elucidate coronary arterial origins or associated defects, and prompt surgical repair. Observation of the exceedingly rare, asymptomatic patient with a small tunnel may be justified by occasional spontaneous closure. All patients require life-long follow-up for recurrence of the tunnel, aortic valve incompetence, left ventricular function, and aneurysmal enlargement of the ascending aorta.

## Disease name and synonyms

In their original description of aorto – left ventricular tunnel, Edwards and Burchell [1] considered the malformation a "separation between the aorta and the heart", or type of aneurysm which "lay against the outflow tract of the right ventricle and origin of the pulmonary trunk". The term "aortico-left ventricular tunnel" was used subsequent to Levy's publication in 1963 [2], and "aorto-left ventricular tunnel" was introduced about ten years later by Ross and colleagues [3]. Recognizing that the tunnel may extend to either the left or the right ventricular cavity, the more general designation "aorto-ventricular tunnels" has recently been applied to this group of malformations [4]. The defect is not a component of any described genetic syndrome, although cystic medial degeneration has been observed in an ascending aortic aneurysm resected fifteen years after tunnel repair [5].

## Definition and diagnostic criteria

**Figure 1:**
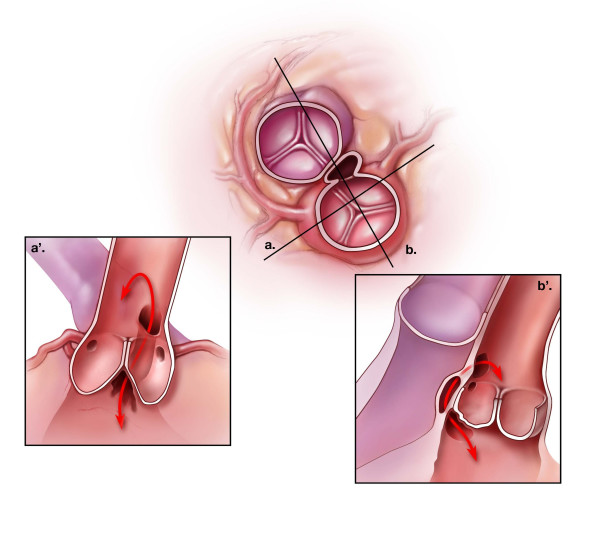
Schematic representation of the most common type of aorto-left ventricular tunnel. The middle figure shows a cross-sectional view at the approximate level of the aortic sinotubular junction. The tunnel passes from the ascending aorta into the tissue plane between the aortic and pulmonary roots. (a') is a longitudinal section across the left ventricular outflow, through the left and right coronary sinuses of Valsalva (plane "a" of the central figure). In this example, the aortic end of the tunnel lies above the ostium of the right coronary artery, while the ventricular end is found within the intercoronary, interleaflet triangle. The position of the aortic opening is variable and may be found anywhere above the left or right coronary sinus, or the intervening commissure. (b') depicts a longitudinal section crossing the noncoronary and right coronary aortic sinuses (line "b" in the central figure). Because the pulmonary valve lies distal to the aortic valve, the tunnel may displace the free-standing, muscular, subpulmonary infundibulum enroute to the left ventricular cavity. It does not, however, pass through any ventricular myocardium.

An aorto-ventricular tunnel is an extracardiac channel which connects the ascending aorta above the sinutubular junction to the cavity of the left or right ventricle. Among 130 cases reported in the literature, more than 90% communicated with the left ventricle (Figure 1). It differs from a ruptured sinus of Valsalva aneurysm (sinus of Valsalva fistula) in having its vascular orifice in the tubular aorta, rather than a sinus of the aortic valve, and in passing outside the heart into the tissue plane between the muscular subpulmonary infundibulum and the aortic valvar sinuses. The aortic opening of most tunnels lies above the right coronary sinus of Valsalva. In these cases, the tunnel virtually always communicates with the left ventricle in the fibrous triangle beneath the left – right coronary commissure, or the right ventricle immediately above or below the subpulmonary infundibulum. In aorto-left ventricular tunnel, the right coronary aortic leaflet is thus unsupported for a variable portion of its hinge-point and may appear to arise from a bar of fibrous tissue spanning the aortic root [5]. Tunnels lying above the left sinus of Valsalva or the intercoronary commissure have less uniform morphology and may enter the left ventricle further away from the aortic valve, apparently through infoldings of fibrous tissue. It is extremely rarely, if ever, that an aorto-ventricular tunnel passes through intracardiac myocardium to reach the cavity of the ventricle, a feature which serves to differentiate it from coronary-cameral fistula [4].

The ostium of a coronary artery may lie within an aorto-ventricular tunnel, and absence of the origin (atresia) of the left [6,7] or right [3,8-13] have both been observed with this anomaly. In one reported case, there was a fistula between the right coronary artery and the distal segment of a tunnel to the left ventricle [11]. Associated lesions of the aortic valve occur in about 20% of patients, ranging from two-leaflet valves without obstruction [1,2] to severe dysplasia or atresia [11,14-16]. In addition, older patients may acquire leaflet perforation [17,18] or aortic incompetence [19] as the result of hydrodynamic trauma to the unsupported right coronary cusp or progressive aortic dilatation. Stenosis of the pulmonary valve [20,21] occurs less frequently (around 5% of reported cases), while compression of the right ventricular outflow tract by the tunnel may produce subpulmonary obstruction [22]. Rarely, both semilunar valves are stenotic [2,23].

Histologically, the arterial end of the tunnel resembles the aorta with fibrous tissue, elastic fibers and smooth muscle cells, while the ventricular end contains hyalinized collagen and muscle. This reflects that the "walls" of tunnels incorporate the structures through which they pass. Within the tunnel itself, there may be a well-defined junction between ventricular and arterial components, in addition to cystic or membranous structures reminiscent of cardiac valve leaflets [4,24].

## Epidemiology

The incidence of aorto-left ventricular tunnel has been variably estimated to be around 0.1% of congenitally malformed hearts from review of clinical and pathological material [25], 0.05% among patients undergoing cardiac catheterization during a 35-year interval at the Children's Hospital in Boston [11], and 0.46% of cardiac malformations identified by fetal echocardiography [5]. About twice as many cases have been reported in males as in females, but it is seldom seen in patients of Asian, Oriental, or African descent. Although extremely rare, aorto-ventricular tunnel is the most common cause of abnormal blood flow from the aorta to a ventricle in infancy.

## Clinical description

A loud "to-and-fro" murmur, usually with systolic and diastolic thrills, invariably radiates over the entire precordium in aorto-ventricular tunnel, and bounding pulses indicate rapid aortic run-off. In older patients, these signs may suggest aortic valve stenosis with incompetence, but the second heart sound should have a normal aortic component in uncomplicated aorto-ventricular tunnel. Although spontaneous closure has been documented by echocardiography in a single case of aorto-left ventricular tunnel [11], most patients develop symptoms of heart failure during the first year of life. The onset, severity and progression of heart failure is, however, quite variable, and ranges from many years of asymptomatic compensation [19,26-28] to rapid decompensation [8,29], sudden death [30], or death *in utero *[16], This spectrum may reflect variable compression of coronary arteries, associated left ventricular outflow obstruction, or obstruction to the right ventricular outflow tract, although it has not, in general, been possible to correlate clinical course with specific morphology of the tunnel. The exceptions to this generalization are aorto-right ventricular tunnel with pulmonary stenosis, and tunnels with severe associated aortic valve obstruction. In the former, the onset of heart failure is delayed [31], while in the latter group, congestive heart failure, with or without low cardiac output, supervenes early, nearly one third of reported cases having died before birth or on the first day of life.

## Etiology

While the etiology of aorto-ventricular tunnel is unknown, the substrate for its formation and that of the associated valvar and coronary arterial lesions may be inferred from developmental anatomy [32-34]. The cushions which form the facing aortic and pulmonary sinuses with their respective valvar leaflets normally become separated by an extracardiac tissue plane, due to regression of surrounding muscle. The coronary arteries, also initially encased by this cuff of myocardium, grow through it to connect with the aortic sinuses. Failure of this tissue plane to develop normally might then result in a tunnel above one of the facing aortic sinuses and explain also the potential involvement of the proximal coronary arteries and valve leaflets. This produces one of the few congenital malformations which may simultaneously involve both the pulmonary and aortic valves.

## Diagnostic methods

Echocardiography is the diagnostic investigation of choice [5,16,35-41]. Transthoracic cross-sectional imaging in a parasternal long-axis view, sometimes with clockwise rotation of the probe [11,39] demonstrates the tunnel itself, as well as its aortic origin and left ventricular opening. Both two-dimensional and real-time three dimensional echocardiography have also established reliable fetal diagnosis [40-42]. On color-Doppler studies, diastolic flow is seen passing from the aorta to the left ventricle, and systolic, from the ventricle to the aorta. Tunnels which open into the right ventricle are visualized in the short axis view, while left ventricular function, which may be variably impaired with hypertrophy and dilatation, is assessed in short axis cuts. Magnetic resonance angiography also has been used to demonstrate tunnels to the left [37] and right [31] ventricles but is not widely available in clinical practice. Cardiac catheterization with angiography is now indicated only when associated lesions or coronary arterial origins cannot be evaluated with certainty on noninvasive studies.

## Differential diagnosis

Aorto-ventricular tunnel must be distinguished from other lesions which cause rapid run-off of blood from the aorta and produce cardiac failure. These include sinus of Valsalva fistula, common arterial trunk with valvar regurgitation, aorto-pulmonary window, ventricular septal defect with aortic regurgitation, persistent patency of the arterial duct, coronary-cameral fistula, valvar aortic stenosis and regurgitation, and cerebral arterio-venous malformation. Because of its "to-and-fro murmur", tetralogy of Fallot with absent pulmonary valve can also mimic aorto-ventricular tunnel with associated right ventricular outflow obstruction.

## Antenatal diagnosis

It is possible to reliably diagnose aorto-ventricular tunnel on fetal echocardiography after 18 weeks gestation. Hypertrophy and dilatation of the left ventricle with progressive reduction of its shortening fraction are consistent features, and there is often disproportionate dilatation of the aortic root with apparent incompetence of the valve. Using color flow Doppler imaging, blood flow around the aortic valve has been demonstrated [16,42], as well as flow specifically within the tunnel itself [40,41]. There are no known molecular markers for aorto-ventricular tunnel at present, and it is not associated with any recognized genetic syndrome. However, the recent finding of cystic medial necrosis within the wall of an ascending aortic aneurysm resected fifteen years after repair of aortico-left ventricular tunnel in early childhood [43] raises the possibility that markers of an associated or underlying connective tissue disorder may emerge.

## Management

In general, surgical correction of a tunnel carrying significant blood flow should be undertaken without delay, even in asymptomatic patients, as only those repaired in the first six months of life have been shown to have subsequent normalization of left ventricular size and function [9]. Based on a single report of spontaneous closure over a two-to-three year period, it has been suggested that observation may be appropriate for the occasional asymptomatic patient with a very small (2 millimeter) aorto-left ventricular tunnel [11]. In this particular case, however, critical valvar aortic stenosis was relieved by balloon valvuloplasty at the time of diagnosis on the first day of life, so extrapolation to other situations should be done with caution.

**Figure 2:**
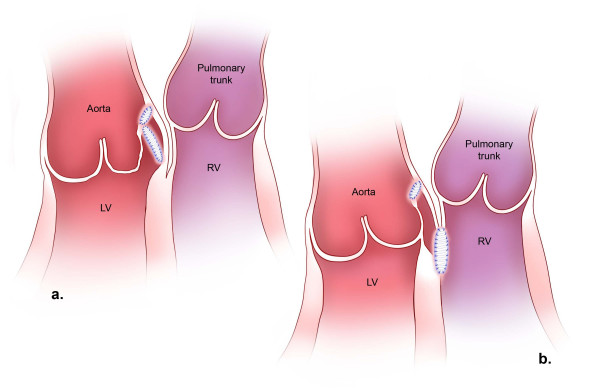
Surgical repair of aorto-left ventricular tunnel (a) and aorto-right ventricular tunnel (b). The aortic orifice of either tunnel is closed with a patch inserted through an aortotomy. A left ventricular orifice is closed with a second patch, placed through the opened tunnel itself, which is then obliterated by reapproximation of its walls over the patch. The upper margin of this second patch attaches to the extra-luminal surface of the first. In the case of aorto-right ventricular tunnel, the right ventricular orifice is approached through the right ventricle or pulmonary valve, and the second patch lies completely separate from that in the aorta.

Repair consists of closing the tunnel such that the aortic valve is supported, the coronary circulation is not compromised, and left or right ventricular outflow obstruction is prevented or relieved. In most cases of aorto-left ventricular tunnel, this has been accomplished by transaortic patch closure of the aortic end, and placement of a second patch through the tunnel itself to close the ventricular orifice and support the aortic valve (Figure 2a). Alternatively, the tunnel wall itself can be used to achieve an equivalent anatomical result [44]. Closure of the aortic orifice by direct suture also has sometimes given good results [11,45,46], but more often, the tunnel recurs or progressive aortic regurgitation through an unsupported or distorted right coronary leaflet leads to subsequent valve replacement. If the ventricular end of an aorto-left ventricular tunnel is not closed, residual high pressure in the blind-ending pouch may compress the right ventricular outflow [22]. In tunnels communicating with a low-pressure right ventricle, it is less certain that the ventricular orifice need be closed, although this has been done through a right ventricular incision in most reported cases (Figure 2b).

When the ostium of a coronary artery arises proximally within a tunnel, the patch is deviated distally to conserve perfusion from the aorta. More distal origin of a coronary artery from a tunnel above the right aortic sinus is managed by resection of the orifice and reattachment to the ascending aorta [12,44,47]. Distal coronary origin in a tunnel arising above the left aortic sinus is more difficult to manage, because it lies behind the heart. As these generally have been associated with tunnels to the right ventricle, however, closure of just the ventricular end is an option to maintain coronary perfusion [31]. Patch angioplasty using autologous pericardium or saphenous vein has been successful in restoring flow to a right coronary artery whose ostium was atretic [7].

Associated lesions of the aortic valve are treated as indicated either separately or at the time of tunnel repair. This has included balloon valvuloplasty [11], open commissurotomy [48-50], homograft root replacement [51], or aortoventriculoplasty [15] for stenosis or atresia in neonates or small infants, as well as repair or replacement of the valve in older patients. Obstruction of the pulmonary valve has been successfully managed by percutaneious valvuloplasty preoperatively [21] or open valvotomy at the time of surgery [23,31]. However, attempted percutaneous balloon dilation did not relieve the obstruction on one occasion [31].

Transcatheter closure of a tunnel to the left ventricle with an Amplatzer duct occluder has been reported in two patients [52,53], but attempted coil closure of one to the right ventricle was not effective [31]. The rationale for avoiding surgery in one patient was coincident noncompaction of the left ventricle with severely reduced left ventricular function. Given the desirability of supporting the aortic leaflet and the variable origins of coronary arteries in this malformation, however, it is questionable if percutaneous interventions can achieve long-term outcomes equivalent to those of current surgical techniques, for which operative mortality approaches zero [9,11].

## Unresolved questions

While follow-up extending to 35 years has now documented that mild aortic regurgitation may remain stable for a considerable period of time in postoperative patients [11,54], the very long-term results of two-patch repair in the modern era are awaited, as are elucidation of the molecular or genetic basis of the anomaly. The natural history of disproportionate ascending aortic enlargement which occurs early in life with this malformation is also uncertain and may eventually emerge as the ultimate determinant of outcome.
